# I’ve been robbed! – Can changes in floral traits discourage bee pollination?

**DOI:** 10.1371/journal.pone.0225252

**Published:** 2019-11-21

**Authors:** Camila Vaz de Souza, Maíra Vidor Salvador, Priscila Tunes, Luiz Claudio Di Stasi, Elza Guimarães

**Affiliations:** 1 Graduation Program in Biological Sciences, Laboratory of Ecology and Evolution of Plant-Animal Interactions, Department of Botany, São Paulo State University (UNESP), Botucatu, São Paulo, Brazil; 2 Undergraduate Course in Biological Sciences, Laboratory of Ecology and Evolution of Plant-Animal Interactions, São Paulo State University (UNESP), Botucatu, São Paulo, Brazil; 3 Laboratory of Phytomedicine, Pharmacology and Biotechnology, Department of Pharmacology, São Paulo State University (UNESP), Botucatu, São Paulo, Brazil; 4 Laboratory of Ecology and Evolution of Plant-Animal Interactions, Department of Botany, São Paulo State University (UNESP), Botucatu, São Paulo, Brazil; INRA-UPMC, FRANCE

## Abstract

Some floral visitors collect nectar by piercing flower external whorls, acting as nectar robbers. They leave robbery vestiges, which can cause changes in floral characteristics, including physical and chemical signals that may influence flower recognition by pollinators. If pollinating bees associate these changes with absence or reduction in nectar volume, they can avoid these flowers, negatively affecting pollination. We aimed to investigate the effect of robbery on primary and secondary attractants. Additionally, we experimentally investigated if the visual signs present in robbed flowers affect the bee pollination of this plant species by discouraging pollinator visits. This study was performed in a very common pollinator-plant-cheaters system comprised by a bee-pollinated Bignoniaceae species and a nectar-robber bee that lands on the corolla tube and makes slits at its base during the nectar robbery. We experimentally isolated the effect of nectar consumption by this nectar-robber and investigated if the slits caused by the nectar-robbers affected the floral scent emission. In addition, we experimentally evaluated the effect of visual signs (slits) associated to the nectar robbery and the effect of nectar depletion on the pollination of *Jacaranda caroba* (Bignoniaceae). The robbers visited around 75% of the flowers throughout the day and removed significant amounts of nectar from them. However, the damages the robbers cause did not affect floral scent emission and we did not verify significant differences on pollen deposition neither when comparing flowers with slits and control nor when comparing flowers with and without nectar. We showed that even though nectar-robbers visually honestly signal the robbery and deplete high amounts of nectar, they did not affect pollinator visitation. These results showed that presumably antagonistic interactions might in fact not be so.

## Introduction

Some groups of floral visitors feed on nectar without pollinating the flowers, and when they cause holes or slits on external whorls, they are named robbers [1]. Many studies investigate the effects of floral nectar robbery on pollination, but they focus mainly on changes in pollinator behaviour due to the reduction in nectar volume [2, 3, 4], which may indirectly affect plant fitness [5].

Flowers rely on chemical and visual sensory signals to advertise their resources [6]. Bees, throughout their lives, learn to use floral cues as predictors of resource and, thus, focus their visits on more profitable flowers [7]. By influence of floral traits that attract pollinators, such as shape, size, colour, scent and flower texture, decision for visitation is made [8]. Additionally, contrasting patterns within a flower are also important cues for flower short-distance recognition by bees [9]. Among all these floral traits, olfactory cues are considered the basis of pollinators’ choice of flowers, especially for bees, that learn faster and remember easier these cues, as compared to their ability to learn visual cues [10]. However, for some bees, visual cues and scent, when combined, may trigger stronger pollinator response than when separated [11]. Additionally, bees can, specifically, detect the presence of floral nectar using olfactory cues [12, 13, 14]. Thus, the floral scent can work as an honest signal, advertising the presence of this trophic resource [10].

What is particularly remarkable about nectar robbers is that they violate floral integrity [1] and leave visual marks on the flowers that might act as honest visual signals of nectar depletion, which could warn the pollinator about nectar unavailability. They can also chemically advertise the robbery if the damage affects the whole floral scent emission [10], or if the nectar itself is signalling its presence through specific compounds [12, 13, 14]. Thus, considering bees’ associating abilities [2] and the ecological benefit of time-place learning [15], we expect that pollinators avoid robbed flowers based on cost-benefit ratio assessment [16], since they warn about nectar depletion, at least visually.

In some plant species, nectar-robbing is remarkably important, especially in those with tubular flowers, which morphology prevents short-tongued visitors to access the nectar legitimately [2]. In fact, the Bignoniaceae family that is known to have tubular flowers, shows an impressive 75% of its interactions established with cheaters, including nectar robbers [17].

In this scenario, we investigated a natural system involving a Bignoniaceae species, which interaction network includes both pollinating bees and bees that rob nectar from its tubular flowers [4]. Thus, our objective was to investigate if, besides visual marks, robbers affect other floral cues and if they do, how? Specifically, we characterized the effect of robbery on the primary attractant, which is the trophic resource nectar, and on the secondary attractants that means chemical and visual signalling. Additionally, we experimentally investigated if the visual signs present in every robbed flower affect the bee pollination of this plant species by discouraging pollinator visits.

## Material and methods

### Study area and study system

The study was carried out at the ‘Estação Ecológica de Santa Bárbara’ (EEcSB), belonging to the ‘Instituto Florestal de São Paulo’ (permit given by the Secretariat for Environment of São Paulo State—SMA 260108–008.158/2014) at the municipality of Águas de Santa Bárbara, São Paulo, Brazil (22°46'- 22°50' S and 49°10'- 49°15' W). The reserve has 2712 hectares of cerrado *sensu lato* (savannah-like vegetation). The humid-dry tropical climate has average temperatures between 16°C and 23°C in the coldest and hottest months, respectively. Annual rainfall is, on average, 1200 mm [18].

In order to answer our questions, we choose a very common biological system in the savannah physiognomies of Brazilian ‘cerrado’, which comprises a Bignoniaceae nectariferous species, *Jacaranda caroba* (Vell.) DC., pollinated by *Bombus morio* Swederus (1787) bee, that have their floral nectar robbed by another bee species, *Oxaea flavescens* Klug. (1807) [4]. This short-tongued bee, specialized in nectar robbing of ‘cerrado’ Bignoniaceae species, presents adaptations in its proboscis that allow a fast and efficient external perforation of the floral tube in order to gather nectar by making slits at the corolla base [19]. Plant samples were registered in SisGen under the number A4DC9A4.

### Floral biology and visitors

We observed three flowers per individual in 15 individual plants (n = 45 flowers) to describe the time of flower opening, duration of anthesis; we also determined stigma receptivity with peroxidase test paper solution [20] and viability of pollen grains with acetic carmine as a vital stain [21]. We performed focal observations of floral visitors [22] with bare eyes, during the light hours of the day, beginning at 7:00 and ending at 18:00 hours. We observed one to two recently-opened flowers per plant in 116 plants, totalling 276 flowers. By the end of the study we had 120 hours of observation, during which we registered the frequency of visits/ flower/ plant/ day, as well as the behaviour of both bees, the nectar robber, *Oxaea flavescens*, and the pollinator, *Bombus morio*.

### Describing the floral attractants in robbed and intact flowers

The slits performed by *O*. *flavescens* are undoubted signs of nectar robbery that could be visually and chemically perceived by pollinators. In order to determine how widespread the signs of nectar robbery were in a natural population, we randomly sampled 276 flowers from 115 plants and described the frequency of slits performed by *O*. *flavescens* in the early morning (08:00 h) and at the end of the day (18:00 h).

#### Primary attractant: Nectar exploitation by robbers

In order to quantify nectar-robbery effect on the total amount of nectar produced per flower, which represents the nectar potentially available to pollinators, we performed a field experiment in which we isolated the foraging effect of *O*. *flavescens* on floral nectar. We randomly took one flower from 57 plants and subjected it to one of two treatments: Robbed flowers–we obstructed the corolla tube entrance with cotton to quantify the exclusive effect of nectar removal by *O*. *flavescens*. We performed this experiment with recently opened flowers and exposed them to visitors for approximately 10 hours. Non-visited flowers: we protected the inflorescences with bridal veil bags to obtain data on the total nectar production per flower without any nectar depletion. Then, by the end of the first day of anthesis, we collected the flowers to perform the nectar measurements. We used calibrated syringes to measure the remaining nectar volume (μL) in each flower from both treatments.

#### Secondary attractant: Chemical signs

We also verified if the slits caused by robbers, *O*. *flavescens*, altered floral scent emission, which potentially could affect pollinator attraction and visitation. In order to check if floral nectar could contribute to the whole floral scent, we sampled control flowers from which we withdrew all the nectar and compared these results with control flowers having all the naturally produced nectar preserved.

For that, we followed the protocol by [23]. We individually enclosed, in polyethylene bags, six recently opened flowers in four plants (n = 1–2 flowers/ plant); two flowers with slits performed by *O*. *flavescens* during nectar robbery (without nectar), two flowers without slits and with all the nectar naturally produced preserved, and two flowers without slits and without nectar, totalling seven flowers. We collected the VOCs accumulated inside the bag with adsorbent traps connected to a membrane pump with airflow of 200 ml/ min. We used adsorbent tubes made from micro vials, filled with a mixture of 1:1Tenax-TA (60–80 mesh) and Carbotrap B (20–40 mesh). We collected each sample during one hour. The samples were stored in a freezer at approximately—20°C prior to the analysis. We analysed the VOCs samples on a Thermo Scientific GC-MS, model FOCUS with an automatic sampler (Thermo–triplus DUO) and coupled to a Thermo–ISQ 230ST mass detector. We used a TG-5MS column and maintained a constant 1 mL/ min flow of helium as the carrier gas. Before the injection, we put the traps in vials for 20 min, at 200°C, in a heating oven. We collected two microliters of sample from inside the vial with an automated gas tight syringe at 140°C and injected immediately into the GC injector in splitless mode, with the injector temperature being 200°C. We co-injected the samples with a 500-μL mixture of n-alkanes (C_7_–C_30_) at 0.1% of concentration. The column temperature was initially 50°C, was increased to 250°C, at a rate of 5°C.min-1, and was kept constant for 5 min after reaching 250°C. The MS interface worked at 250°C. The detector was operated in electron impact ionization mode (70 eV), with a scanning range of 34–350 m/z. In order to try identifying the volatile compounds we used NIST 08, and [24] mass spectral libraries. Besides, we compared the mass spectra and Kovat’s Retention Indices of target compounds with that of authentic standard compounds. For quantitative analysis of floral volatiles, we injected 100 ng each of ca. 150 components into the GC-MS system. Among them, there were monoterpenes, aliphatic, and aromatic compounds. To estimate the total amount of scent available in the scent samples, we used the mean of the peak areas (total ion current) of these compounds [25]. Additionally, during the field experiments to test the ‘effect of nectar-robbery and visual signs on pollinator visitation’, we performed the same analysis of floral volatiles with three recently opened flowers (n = 3 plants) that had manually-made slits, in order to control the variable scent in all the treatments of the experiment described below. We verified that the scent emitted by flowers with manually-made slits did not differ from the scent emitted by flowers from the other treatments (PERMANOVA; Pseudo-F = 0.49368, p = 0.7418).

### Experimentally testing the effect of nectar-robbery on bee-pollinator visitation

In order to evaluate the effects of the visual signs (slits) associated to the nectar depletion by the nectar robber, *O*. *flavescens*, on the pollination of *J*. *caroba* flowers, we performed field experiments in three consecutive years (2017–2019). We manually caused the damages to the flowers at the moment of flower opening, just before the assemblage of the experiment in the field. The slits were made with a sharp needle and were identical to those caused by *O*. *flavescens*, with the same shape, size and placement on the flowers (upper portion of the corolla tube basis). Additionally, in order to check if the presence of nectar could interfere on pollinator choice, we also included this variable in the experimental design. We ensured that we had flowers with all of the nectar removed and flowers with all the nectar preserved in both treatments, ‘*Experimentally damaged flowers*’ and ‘*Control flowers*’. We performed these experiments by manually manipulating the nectar and the slits, in order to standardize potential sources of interference on the experiment’s results. This means that the flowers from all the treatments were virgin, recently opened, exposed to floral visitors at the same time and emitted similar scent during the experiments.

**Fig 1:**
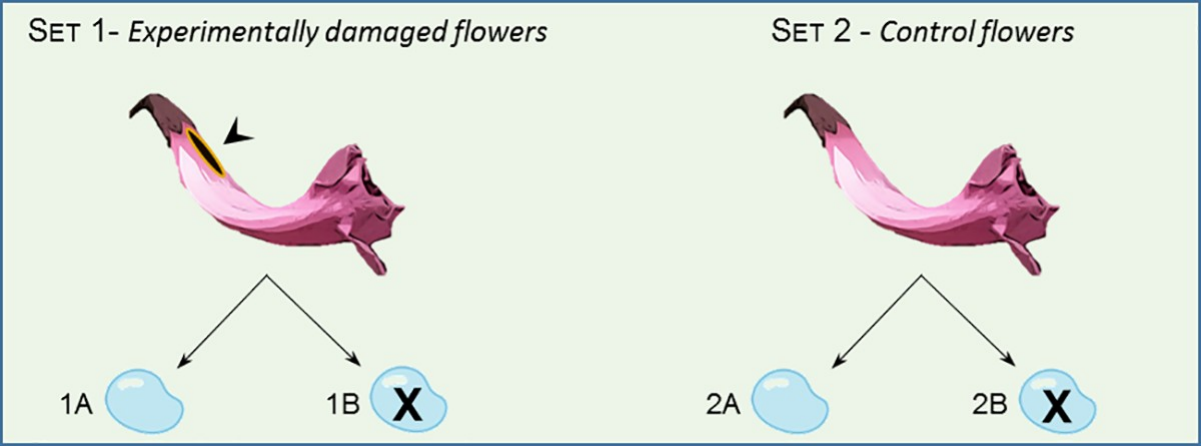
Experimental design to test the effect of nectar-robbery on bee-pollinator visitation. Set 1 - (1A) Experimentally damaged flowers with nectar, (1B) Experimentally damaged flowers without nectar. Set 2 –(2A) Control flowers with nectar, (2B) Control flowers without nectar.

We randomly took a total of 150 flowering individuals (N_flowers_ = 230, varying from 1 to 2 flowers per plant), and divided them into two sets of treatments based on the presence/ absence of manually-made slits (Fig 1):

In the set 1, ‘*Experimentally damaged flowers*’, we used flowers with manually-made slits simulating the visual signs of robbery, (1A) *Experimentally damaged flowers with nectar*: 60 flowers (1–2 per plant, 40 plants) with slits, but with the nectar preserved and (1B) *Experimentally damaged flowers without nectar*: 58 flowers (1–2 per plant, 36 plants) with slits, but with all the nectar withdrew with the help of a micro syringe.

In the set 2, ‘*Control flowers*’, we used flowers that had no visual signs of nectar robbery, (2A) *Control flowers with nectar*: 57 flowers (1–2 per plant, 39 plants) without any slit, with the nectar preserved and (2B) *Control flowers without nectar*: 55 flowers (1–2 flowers, 35 plants) without any slit, but with all the nectar withdrew with the help of a micro syringe.

In both sets of treatments, to ensure that undamaged flowers (2A) and (2B) remained intact from the beginning of their opening until the end of the experiment, we wrapped the corolla basis with a transparent matte tape. We also coated the flowers with slits with the same transparent tape (1A) and (1B) to standardize and avoid any bias that could have been caused by it on pollinator visitation. All the flowers remained exposed to floral visitors for 10 hours. By the end of the exposure period, we collected the stigmas from all flowers and fixed them on slides containing acetic carmine solution. Then, we registered the presence/ absence of pollen grains deposited onto the surface of each stigma under a light microscope.

### Statistical analysis

We evaluated the probability of a bee visiting flowers with and without slits by modelling the probability of pollen grains (binary variable) being deposited onto the stigmas of each treatment, using GLMM with binomial error distribution, considering treatment as a fixed factor, individual plant and sampling year as random variables. Hereinafter, we will refer to this as the probability of a flower receiving a pollinator visit, as we only used the pollen grains deposited per flower as a proxy of legitimate pollinator visits. We verified *a priori* that the data did not differ between years and that the statistical models performed with each year separately showed similar results to the final model comprising all three sampling years (Table 1), thus, our final model comprised all the sampling years.

**Table 1:** Structure and results of the GLMM with binomial error distribution performed in this study. We performed GLMM with binomial error distribution to compare the probability of a bee visiting flowers with and without slits and with and without nectar within these treatments. As our data set was collect in three consecutive years, we verified *a priori* that the data did not differ among years by performing the same analysis with the data from each year individually. Here, we show the structure of the models used and the statistical results obtained for each year. These models showed similar results to the final model comprising all the years. Thus, we used for the final analysis our complete dataset, comprising all the sampling years, and added the sampling year as a random variable in the model.

	GLMM structure	Results	
		Stats value	p-value
mod.year1	(probability ~ treatment + (1|plant)	Z = -0.503	0.615
mod.year2	(probability ~ treatment + (1|plant)	Z = -0.121	0.903
mod.year3	(probability ~ treatment + (1|plant)	Z = 0.145	0.885
mod.all.years	(probability ~ treatment + (1|plant) + (1|year)	χ^2^ = 0.414	0.937

We compared the amount of nectar remaining in the flowers after a full day of foraging exclusively by nectar-robbers to the total daily nectar production of *J*. *caroba* flowers by performing Mann-Whitney U test.

We performed permutation analysis of variance (PERMANOVA) to verify if there were any statistical differences between the scent emitted by flowers of *J*. *caroba* that had been naturally damaged by *Oxaea flavescens* (with robbery slits and without nectar) and control flowers with and without nectar. Additionally, we used non-metric multidimensional scaling (NMDS) to graphically display the floral scent from these flowers, based on Bray-Curtis dissimilarity. We performed the statistical analyses using R v. 3.5.2 [26] with standard and additional packages: lme4 [27], nlme [28] and vegan [29] and Primer 6 v. 6.1.15 with PERMANOVA+ v. 1.0.5 [30].

## Results

### Floral biology and visitors

The flowering of *J*. *caroba* occurred from July to October and each flower was functional for at least four days. Although the beginning of anthesis was concentrated in the early hours of the day, around 7:00 h, we observed some flowers that opened throughout the day. The stigmas were receptive and showed opened lobes at the moment of flowers opening and remained receptive until at least the second day of anthesis; 95.7 ± 2.96% of the pollen grains were viable.

**Fig 2:**
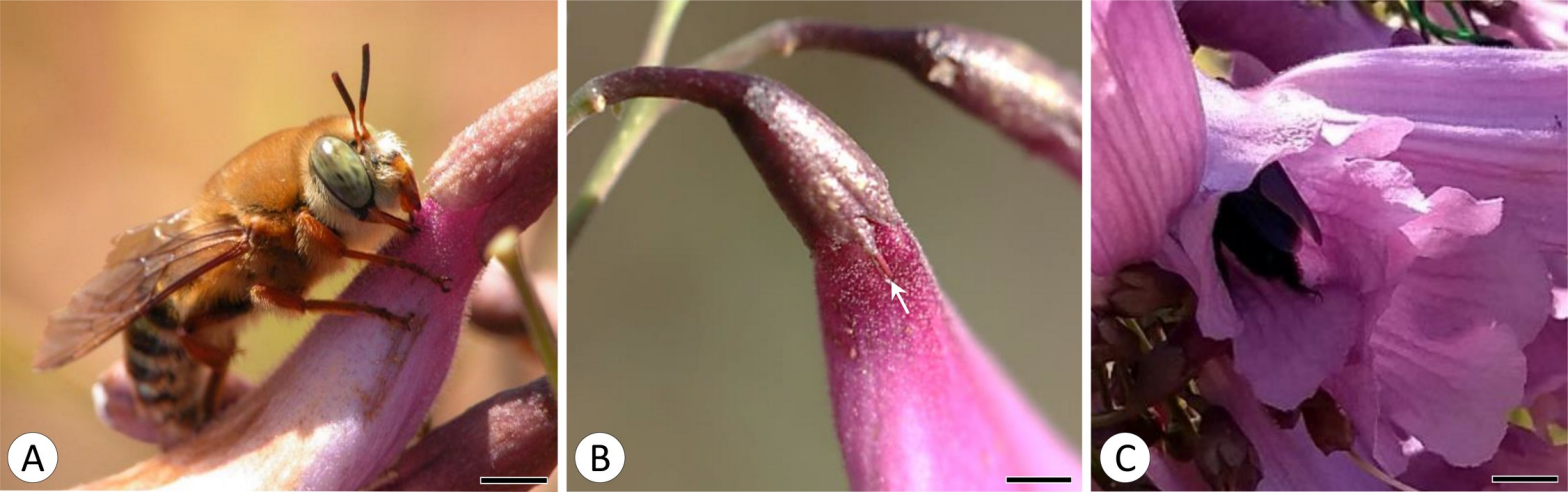
Floral visitors and robbery sign in *Jacaranda caroba* flowers (Bignoniaceae). (A) *Oxaea flavescens* robbing nectar from a *J*. *caroba* flower. (B) Slit caused by *O*. *flavescens* in a flower of *J*. *caroba*. (C) *Bombus morio* legitimately visiting a *J*. *caroba* flower. Scale bar: 0.5 cm.

*Oxaea flavescens* always behaved as a nectar robber. This Andrenidae bee landed on the floral tube with its head facing the calix and pierced a slit through the corolla tube base (Fig 2A), which allowed it to access the nectar accumulated in the nectariferous chamber. It left one or two slits per visit at the upper portion of the corolla basis (Fig 2B), which means that the slits were visible to pollinating bees when they were flying and approaching the flowers. *Oxaea flavescens* visited approximately three flowers in one or two inflorescences per plant per visiting round, remaining for approximately two seconds in each flower. Its visits began at about 8:30 h, with a peak of visitation from 10:00 h to noon. The average frequency of visitation was 0.68 ± 0.66 visits/ flower/ plant/ day. Regarding pollinating bees, in the two years of field observations, we registered only *B*. *morio* legitimately visiting *J*. *caroba* flowers by entering the floral tube searching for nectar (Fig 2C). Its peak of visits occurred from 8:00 h to 9:00 h with an average frequency of 0.04 ± 0.07 visits/ flower/ plant/ day.

### Describing the floral attractants in robbed and intact flowers

In relation to the intensity of nectar-robbery performed by *O*. *flavescens*, we observed that at early morning, at 7:00 h, only 8.69% of the flowers presented slits. However, from 8:30 h on, the frequency of visits of *O*. *flavescens* increased, so that by the end of day, 73.18% of *J*. *caroba* flowers had signs of nectar robbery.

#### Primary attractant: Nectar exploitation by robbers

**Fig 3:**
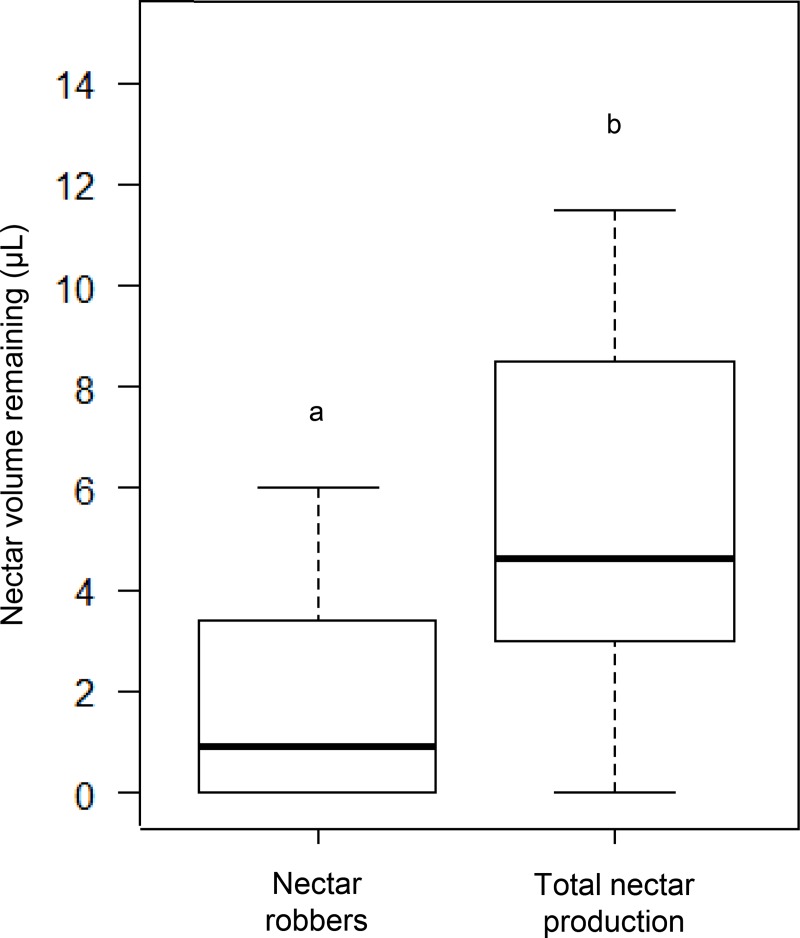
Nectar volume remaining in flowers of *Jacaranda caroba* (Bignoniaceae) after the ‘Nectar exploitation by robbers’ experiment. Volume of nectar remaining in the flowers of *Jacaranda caroba* that had been exposed for approximately 12 hours to exclusive nectar-robber visits, and in the flowers that had been protected from any visitor (total nectar production). The box plots show the median (horizontal line across the box), 25th and 75th percentiles (lower and upper edges of the box) and the upper and lower whiskers, which correspond to the higher and lower data that is no further from the box than 1.5 times the interquartile range. Any data lying beyond the whiskers was considered an outlier (empty circles).

Nectar production in flowers of *J*. *caroba* started in the pre-anthesis period and lasted at least until the second day of anthesis. We found that the nectar remaining in the flowers visited exclusively by *O*. *flavescens* differed from the total nectar produced per flower (U_1,54_ = 56, p < 0.0001) (Fig 3). Robbed flowers showed a median of 0.9 μL of nectar (0.0–6.0 μL, min-max) and the total amount of nectar produced per flower was 4.6 μL (0.0–11.5 μL, min-max).

#### Secondary attractant: Chemical signs

We identified six volatile organic compounds (VOCs) in the dynamic headspace scent samples of *J*. *caroba* flowers, mainly alkanes, ketones and benzenoids (Table 2).

**Table 2:** Total number of compounds, mean of total scent emitted per flower and median (min–max) of relative amount of floral scent emitted by *Jacaranda caroba* (Bignoniaceae) flowers. Naturally damaged flowers corresponds to flowers that were sampled in field with damages caused by nectar-robber bee, *Oxaea flavescens*, control flowers with and without nectar were intact flowers used as control, and experimentally damaged flowers correspond to flowers that had manually-made damages (slits), simulating the visual signs of nectar-robbery (N_total_ = 6 flowers, from 5 plants).

	RI	Naturally damaged flower	Control flower		Experimentally damaged flower
		without nectar(n = 2)	with nectar(n = 2)	without nectar (n = 2)	with nectar(n = 3)
**Total of compounds**		6	6	6	6
**Mean total scent emitted per flower (ng/h ± SD)**		150.57 ± 46.44	29.90 ± 22.48	122.73 ± 28.62	58.24 ± 11.39
**Compounds**^a^		**Median amount emitted per flower (ng/ h)(Min—Max)**			
**FATTY ACID DERIVATIVES**					
***Alkanes***					
Tetradecane	1400	29.42 (26.26–32.57)	8.64(6.51–10.76)	27.91 (19.34–36.49)	10.78 (8.95–11.71)
Hexadecane	1600	35.22 (27.5–42.93)	10.18 (4.84–15.52)	29.98 (15.89–44.06)	11.7 (11.26–16.16)
***Ketones***					
2-Tridecanone	1496	34.19 (20.91–47.46)	6.80 (3.51–10.09)	21.71 (15.77–27.65)	10.81 (7.13–11.3)
2-Pentadecanone	1697	19.86 (18.66–21.05)	3.65 (0.00–7.31)	19.52 (12.22–26.81)	8.32 (4.11–8.98)
**BENZENOIDS**					
Benzaldehyde	960	22.73 (6.47–38.99)	11.94 (10.3–13.6)	16.39 (12.24–20.53)	14.56 (2.88–28.43)
Methyl salicylate	1191	9.17 (1.94–16.39)	2.83 (2.71–2.94)	7.23 (4.1–10.36)	2.2 (0.92–4.51)

RI. Kovat’s retention index; SD. standard deviation.

^a^ Compounds within classes are listed according to Kovat’s retention index

### Experimentally testing the effect of nectar-robbery on bee-pollinator visitation

**Fig 4:**
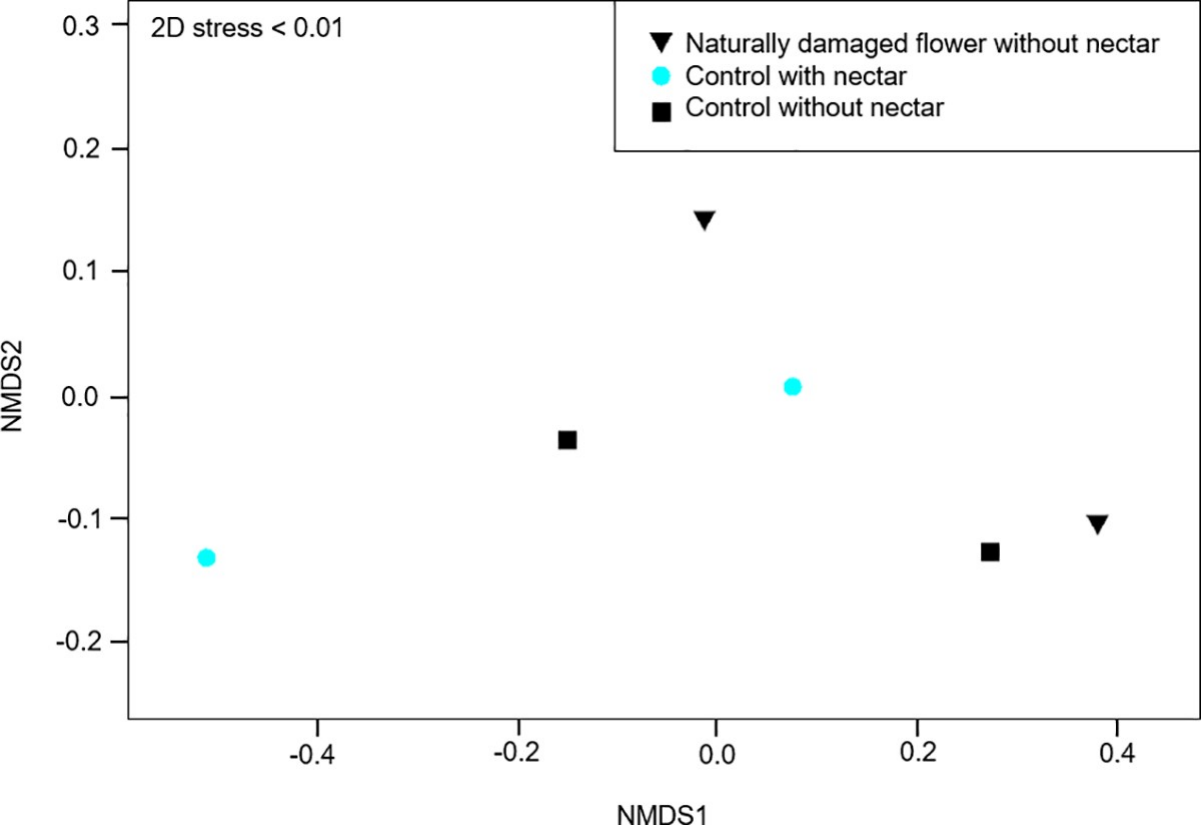
Non-metric multidimensional scaling (NMDS) of scent samples from *Jacaranda caroba* flowers with and without nectar-robbery damages. Non-metric multidimensional scaling based on Bray-Curtis similarities.

We verified that the absolute amount of each compound present in scent did not vary significantly among flowers with and without manually-made slits (PERMANOVA, Pseudo-F = 0.7093, *p* = 0.5302) and non-metric multidimensional scaling showed an overlap of both treatments in *J*. *caroba* (Fig 4). These results showed that manually-damaged flowers do not statistically differ in scent composition from intact flowers. This means that, during the field experiments, the variable scent was controlled.

**Fig 5:**
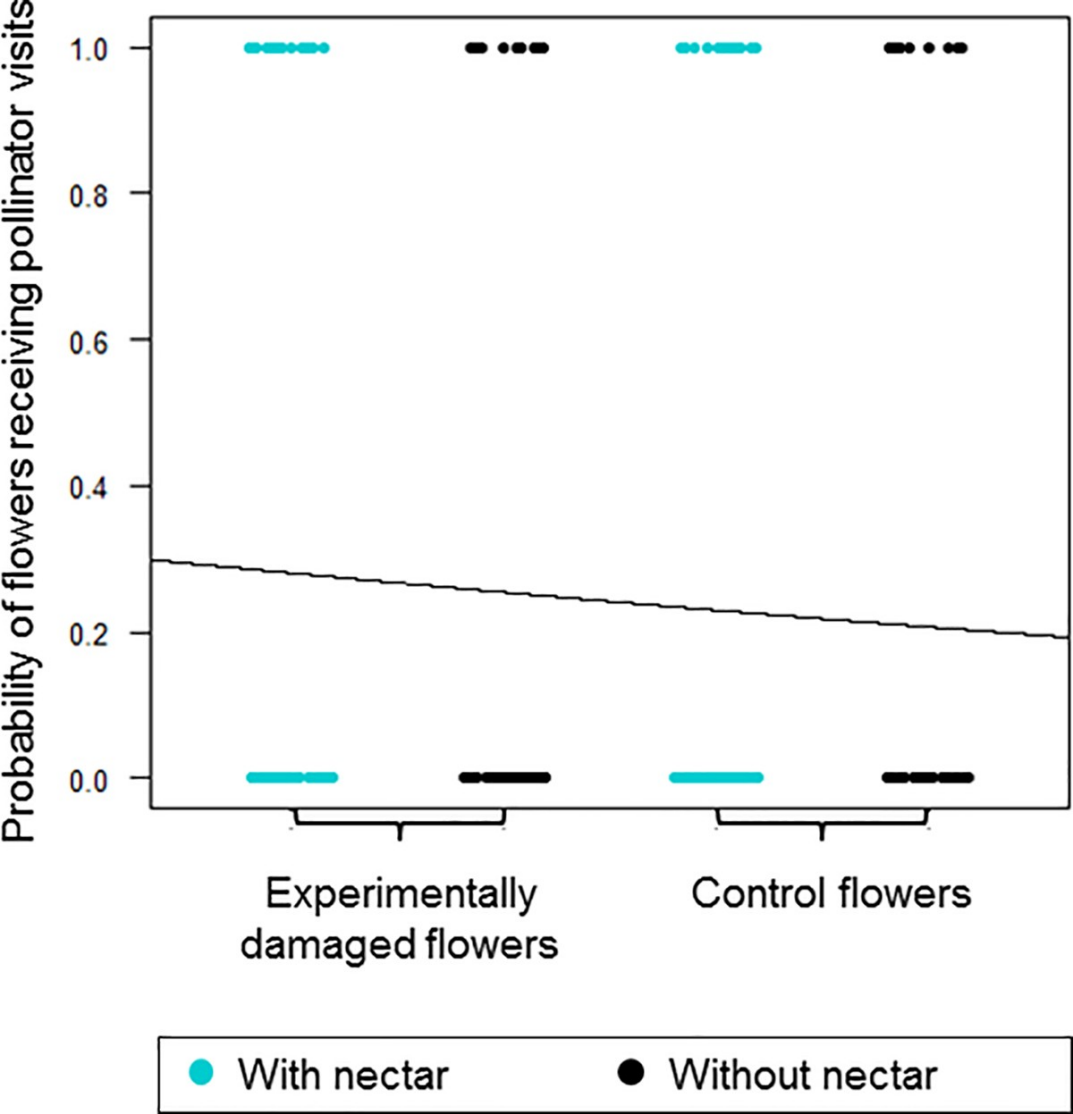
Probability of *J*. *caroba* flowers receiving pollinator visits in both *‘Experimentally damaged’* and *‘Control’* treatments with and without nectar.

There was no statistically significant difference regarding the probability of *‘Experimentally damaged flowers’* with or without nectar and *‘Control flowers’* with or without nectar of *J*. *caroba* receiving pollinator visits (χ^2^ = 0.414, *p* = 0.937, N_flowers_ = 230, N_plants_ = 150) (Fig 5).

## Discussion

In this study, we characterized the changes in floral features elicited by the nectar robbers. We found significant changes in the amount of nectar available to pollinators and a high frequency of flowers with visual signs of robbery in the population. However, we did not detect significant changes in floral scent emission after *O*. *flavescens* had robbed the flowers. We also, registered no change in the scent emitted by flowers after the manually-made slits. We verified, as well, that nectar presence/ absence did not alter floral scent profile. This finding was important because it allowed us to explore, the isolated effects of both the visual signals provided by the slits and the nectar depletion, without the interference of any chemical signs alteration. Therefore, in the experiments performed under natural conditions, we found that the pollinator of *J*. *caroba* visited flowers indiscriminately, despite the changes in visual signs or in nectar volume, both associated to the intense foraging of *O*. *flavescens*. A previous study with the same plant-bee system showed no effect of natural damages on pollen transfer [4]. This finding elicit some questions as: besides affecting a primary attractant (resource), nectar robbers might also change secondary attractants (visual and chemical signs)? If so, to which kinds of floral changes these pollinators were not responding?

The encounter of flowers by anthophilous animals depends on communication mechanisms that are enhanced when floral traits are more suited to animals’ sensory abilities [31]. Thus, the sensory perceptions of pollinators could be important in driving complex pollinator-plant-cheater systems. In this context, nectar robbery may cause various effects on plant reproductive success [2, 32]. A possible negative consequence of the decrease in pollinator visits to robbed flowers would be that, in an extreme scenario, it could lead the pollinator to abandon this species and start to explore another plant species, negatively affecting plant reproductive success, even in non-robbed plants. However, the process of learning new rewarding flowers requires time investment and a high-energy cost [33, 34], which could explain why *B*. *morio* indistinctly visited flowers with and without damage, and with and without nectar.

Additionally, it has been demonstrated that medium-sized bees could have less ability to avoid robbed flowers, not ceasing to visit flowers with low amounts of nectar [35, 36, 37]. When a pollinator is foraging for trophic resource in nature, it always deals with a scenario of uncertainty regarding nectar availability, since the flower might have been previously visited by another pollinator or by nectar cheaters. We could expect that when nectar-robbing is frequent, pollinators are probably used to face a lower volume of nectar standing-crop. In this case, pollinators may need to increase the number of flowers they visit to compensate the low nectar availability per flower and to meet their daily metabolic requirements, thus, favouring pollen flow [2]. This is especially important in self-incompatible plants [36, 38], as is the case of *J*. *caroba* (unpublish. data). In this scenario, the increased flight distance among plants that might occur when bees face robbed flowers [39], may improve the chances of pollen from a plant reaching the stigmas of a compatible mate.

Additionally, according to the idea of optimal meal size, highly mobile animals, such as some medium-sized bees, could optimize their rate of net energy intake by not carrying too much extra weight due to the ingestion of a large meal [40]. This and other optimality hypothesis explain that animals consume only a portion of the available food, as the animal’s rate of net energy intake is negatively associated to the amount of time spent feeding [41 and references therein]. Thus, if a pollinator visited a flower that had been previously visited by the nectar-robber *O*. *flavescens*, it would probably completely withdraw the remaining floral nectar and fly to visit another flower. This scenario could even be favourable for pollinators if the remaining nectar volume, after *O*. *flavescens* visits, is large enough to surpass the energetic cost of a visit, and small enough not to overweight the bees during flight. Therefore, in self-incompatible species the activity of nectar robbers might end up being positive for both mutualistic partners, pollinators and plants, by reducing the cost of flight for bees and increasing the chances of compatible pollen transfer for plants.

Additionally, bees are capable of remembering the times of day in which a given patch of flowers was more profitable [7, 15], thus, it would be expected that the visitation patterns of bees throughout the flowering period reflected this knowledge. In fact, when observing the moments of the day when *O*. *flavescens* and *B*. *morio* visit *J*. *caroba*, we recognize that the pollinators forage before the peak of *O*. *flavescens* activity, as shown by [4]. However, in our study system, we found that the foraging period of both nectar-robbers and pollinators was not related to how pollinators reacted to the visual signals of robbed flowers. As we showed experimentally here, even when the slits, simulating nectar-robbery, were present before pollinator activity, they did not affect pollinator visitation. Therefore, this finding showcases that the slits are not perceived as a sign that discourages pollinator visitation.

Floral signs are usually assumed to be correlated with floral resources, providing ‘honest’ signals for pollinators [10, 42, 43]. Thus, the visual damage left by *O*. *flavescens* could act as an honest signal of nectar robbery, which could be used by floral visitors as a decision-making cue during foraging. Nevertheless, the pollination mutualism is persistent in *J*. *caroba*, even when these ‘honest’ visual signals are present, announcing nectar exploitation by nectar robbers, as reported for other systems [2, 44].

The absence of pollinator response to nectar-robbery may be related to the important role of floral volatiles in directing animals to flowers [31, 45, 46]. The ability to associate floral scent with the presence of resource has an even greater significance in the case of pollinators that present high olfactory acuity, such as bees [10]. In a study about bumblebees’ floral visit constancy, using multiples signals, it was shown that these bees had great difficulty to distinguish between flowers with similar scents even though the flowers had different visual cues [47]. Moreover, specific scents can trigger bees’ spatial memory [7]. Thus, the fact of *J*. *caroba* did not present differences in floral scent composition may represent a key factor for *B*. *morio* to visit both robbed and intact flowers. Additionally, flowers with and without damage emitting the same chemical compounds may prevent pollinators from learning and avoiding the absence or scarcity of resource based on chemical cues [10]. Therefore, in this scenario, tubular flowers with hidden nectar production, as *J*. *caroba* flowers, may benefit from not being completely honest, because pollinator visits remain constant. In addition, as another *Jacaranda* species [48], *J*. *caroba* may replenish the nectar throughout the anthesis, which can make flowers worth the risk for pollinators. And also, since *J*. *caroba* nectar does not signal its presence by its own chemical cues, as other species do [12, 13, 14], pollinators do not have a clear information about nectar content in this system.

There are many examples in the literature showing that nectar robbing may influence multiple mechanisms at the same time, being able to positively and negatively affect pollinator behaviour and, consequently, plant species reproductive success [2, 49, 50, 51, 52]. However, the impact of robbers on secondary floral attractants has been poorly explored. In this scenario, our study brings a broad set of information concerning the effects of the most potentially insidious of the cheaters, the robbers, on floral attractants. Among all the cheaters, they are the ones that are more prone to promote significant changes in floral attractants, since they violate floral integrity, which may trigger a cascade of plant responses. We showed that even though nectar-robbers visually honestly signal the robbery and deplete high amounts of nectar, they do not affect pollinator visitation. Therefore, this seemingly antagonistic bee is, in fact, not antagonistic at all. Our findings suggest that this system may be resilient to nectar-robbery and may sustain not only the pollinators, but also the robber bees through a complex and subtle balance involving trophic resource supply to both groups of floral visitors.
